# P-1902. Self-Reported Impact of Group-Based Coaching During Infectious Diseases Fellowship

**DOI:** 10.1093/ofid/ofaf695.2071

**Published:** 2026-01-11

**Authors:** Mariam Aziz, Laura N Hernandez Guarin, Ami Shah

**Affiliations:** Rush University Medical Center, Chicago, Illinois; Rush University Medical Center, Chicago, Illinois; Rush University Medical Center, Chicago, Illinois

## Abstract

**Background:**

There is a mismatch between the demand for Infectious Diseases (ID) physicians and trainees’ interest in the field. As part of the 2023 IDSA Workforce Development Strategy, the Society emphasizes the need to foster career development opportunities in all levels of training. Coaching during training is well received, helps build skills beyond clinical knowledge, and may prevent burnout. Formal coaching strategies in the literature are longitudinal and individual. Small group coaching is a novel, scalable intervention that also fosters community. We describe our experience with group coaching sessions from 2023 to 2024.

**Methods:**

Four 1.5-hour sessions were offered; 10 fellows participated in each session. Small group coaching, starting with a short topic presentation, was followed by a guided discussion led by a trained physician coach. Sessions occurred in non-clinical, comfortable settings over meals. Topics included “What is your why?” “Setting Boundaries,” “Work-Life Balance,” and “Imposter Syndrome.”

**Results:**

In November of 2024 a RedCap survey was distributed to 20 fellows (classes 2023 – 2026). 18 responded, 13 had done at least one coaching session. 77.8% of the participants didn’t have any professional coaching prior to enrolling in our fellowship. 83.3% noted that before coaching their career goal matched their life goals, 66.7% felt they had not achieved a life-work balance. 50% of the respondents felt they’ve had support to design their career.

After coaching 92% noted that they had tools to align their career and life goals, 85.7% felt they had the tools to have a life-work balance, and 92% had the tools to set up boundaries. 92% liked the group coaching approach.

Useful themes included: Imposter syndrome, career design, work-life balance and charting.

Limitations: Group dynamics and ensuring psychological safety may be important to ensure engagement.

**Conclusion:**

Group coaching during fellowship promotes well-being and alignment of personal growth and career goals. It may reduce burnout through reflective practice, peer validation, feeling more confident in navigating complex team dynamics, and helping to shape a professional identity beyond clinical competency, while fostering a growth mindset and constructive approaches to failure.

**Disclosures:**

All Authors: No reported disclosures

